# Body Composition in Children and Adolescents Residing in Southern Europe: Prevalence of Overweight and Obesity According to Different International References

**DOI:** 10.3389/fphys.2019.00130

**Published:** 2019-02-19

**Authors:** Guillermo Felipe López-Sánchez, Maurizio Sgroi, Stefano D’Ottavio, Arturo Díaz-Suárez, Sixto González-Víllora, Nicola Veronese, Lee Smith

**Affiliations:** ^1^Faculty of Sports Sciences, University of Murcia, Murcia, Spain; ^2^Faculty of Medicine and Surgery, University of Rome Tor Vergata, Rome, Italy; ^3^Teacher Training Faculty of Cuenca, University of Castilla-La Mancha, Cuenca, Spain; ^4^Laboratory of Epidemiology and Biostatistics, National Institute of Gastroenterology-Research Hospital, IRCCS “S. de Bellis”, Castellana Grotte, Italy; ^5^Cambridge Centre for Sport and Exercise Sciences, Anglia Ruskin University, Cambridge, United Kingdom

**Keywords:** fat mass, BMI, nutritional status, WHO, IOTF, CGF

## Abstract

The objective was to analyze body composition in children and adolescents of Southern Europe to identify prevalence of overweight and obesity. This investigation involved 512 girls and 488 boys between 7-to 19-years. Variables evaluated were Body Mass Index (BMI) and Fat Mass (FM; electrical bioimpedance). The references used to establish prevalence according to BMI were those of the World Health Organization (WHO) and the International Obesity Task Force (IOTF); in the case of FM, the Child Growth Foundation (CGF) reference was used. There were significant differences (*p* < 0.05) in the prevalence of overweight and obesity between the three classifications (32.3% according to IOTF, 37.3% according to WHO, and 39.8% according to CGF), being higher in males. WHO-IOTF concordance was substantial (kappa = 0.793), whereas concordances WHO-CGF (kappa = 0.504) and IOTF-CGF (kappa = 0.447) were moderate. The authors recommend evaluating overweight and obesity not only with BMI, but also with FM, and always specify the references used.

## Introduction

Overweight and obesity can be defined as an abnormal or excessive accumulation of fat that can be harmful toward one’s health (World Health Organization [WHO], 2017). Body mass index (BMI) is a simple indicator of the connection between weight and height that is frequently used to indirectly identify overweight and obesity (World Health Organization [WHO], 2017). The simplicity and low cost of BMI has made it a popular indicator to identify overweight and obesity in science and practice (McCarthy et al., 2006). However, BMI does not distinguish between increased mass in the form of fat, lean tissue or bone, and consequently, it may lead to significant misclassification. Therefore, due to excess fat being a pathology that defines obesity, it would be ideal to also evaluate total fat mass (McCarthy et al., 2006). The evaluation of fat mass *per se* allows one to obtain important information about the state of the health of the population under study, as well as identify those at risk of certain diseases (Alburquerque Sendín, 2008). For example, excessive fat mass has been shown to be associated with Type 2 Diabetes Mellitus (Abdullah et al., 2010), cancer (Renehan et al., 2008), coronary heart disease and associated risk factors (Bogers et al., 2007), depression (Luppino et al., 2010), and early mortality (Flegal et al., 2013), to list just a few.

Hence, it is clear when studying the prevalence of overweight and obesity, that it is highly recommended to evaluate not only BMI, but also the percentage of fat mass. In addition, consideration must be given to the cut off points used to classify children and adolescents, an aspect dealt with in previous studies (Wang and Wang, 2002; De Onis and Lobstein, 2010; Shields and Tremblay, 2010; Espín Ríos et al., 2013; Bergel et al., 2014; Lasarte-Velillas et al., 2015; Polo Martín et al., 2015). These studies investigated the prevalence of overweight and obesity but focused only on the cut-off points for BMI and not on cut-off points for fat mass. The present article adds to this literature by comparing cut-off points for BMI but also for fat mass, studying the three main international references: World Health Organization (WHO), International Obesity Task Force (IOTF), and Child Growth Foundation (CGF).

The main objective of this research is to evaluate the BMI and fat mass of children and adolescents residing in Southern Europe, studying the prevalence of overweight and obesity according to common international references of these two indicators, and observing the degree of concordance that these different classifications present. This will provide updated data on BMI, fat mass, and prevalence of overweight and obesity in children and adolescents residing in Southern Europe. Moreover, this study will provide evidence about differences between common international references when classifying children and adolescents weight status.

## Materials and Methods

### Sample

A total of thirteen schools from Southern Europe, nine from Southern Italy (regions of Lazio and Calabria) and four from Southern Spain (region of Murcia) were included in this study. The final sample was made up of 1,000 children and adolescents (512 female and 488 male) between the ages of 7 and 19 years. Excluded from the study were those children and adolescents who did not fulfill any of the recommendations for an adequate analysis of electric bioimpedance, described below. The estimated maximum sampling error at 95% confidence level (*p* ≤ 0.05) for a sample size of 1000 is ±3.1% (Wimmer, 2011).

This research project was carried out according to the International Code of Medical Ethics (Declaration of Helsinki) for experiments with human beings and, was approved by the Research Ethics Commission of the University of Murcia (No. 03/02/2012). Moreover, the parents/legal guardians of all the participants signed an informed consent form for their children and adolescents to take part. Also, children and adolescents provided assent. The children and adolescents were coded individually, and the details treated anonymously.

### Analysis of Body Composition

The measurements were carried out at school, in an indoor hall prepared for the occasion during the morning timetable. Height was measured with the portable height rod of Tanita model Leicester HR 001 (Tanita, Tokyo, Japan), with the precision of 0.1 cm and with the subjects standing up and barefooted. Weight and total fat mass were measured with the Tanita BC-418-MA Segmental Body Composition Analyzer (Tanita, Tokyo, Japan), with the corresponding correction for the weight of the clothes (underwear or short sleeve). The procedure required the subjects to be standing with bare feet on the places marked on the analyzer, at the same time as they held onto the handles, one in each hand. The analysis through electric bioimpedance lasted approximately 30 s per subject. BMI was calculated with the formula Weight (kg) / Height^2^ (m). Even though the Tanita BC-481-MA Analyzer provides separate measurements for the fat in the torso and the inferior and superior extremities, only the percentage of total fat mass was taken into consideration to analyze the prevalence of overweight and obesity in the study sample. As indicated by McCarthy et al. (2006), the equations used for this model are based on bioimpedance, weight, height and age, and were obtained through calibration and validation studied with Dual-energy X-ray absorptiometry (DXA) and BodPod, having a standard error of 2.7% for the body mass of boys and of 2.8% for girls. The validity of this method was also established by the studies carried out by Merritt and Ballinger (2003) and Prefontaine and Ballinger (2003). The software used to pass the data to the computer was Suite Biologica 7.1. Moreover, all recommendations to collect electric bioimpedance data were followed (Sgroi and De Lorenzo, 2011; TANITA, 2016). First, a letter was given to parents, teachers and participants explaining in detail procedures that had to be followed before data were collected, such as no excess of food and drink the day before, no intense exercise in the last 12 h, no alcohol consumed in the last 12 h, no metallic objects, no pace-makers, not done during the menstrual cycle and not during pregnancy. These aspects were also checked by researchers asking participants prior to data collection. Data were collected at 11.00 am, before lunch, to ensure that data were collected more than 3 h after participants woke, and to avoid participants eating and drinking 3 h prior to measurement. Finally, prior to data collection the participants were asked to urinate to follow standard procedure for biompedance measurement.

### Studies Used as References

The references used to establish the prevalence of overweight and obesity according to the BMI were from the WHO (De Onis et al., 2007) and the IOTF (Cole et al., 2000; Lobstein et al., 2004; Cole and Lobstein, 2012); in the case of FM, CGF was used as a reference (McCarthy et al., 2006).

### Statistical Analysis

First, the normality of continuous variables was assessed through the Kolmogorov-Smirnov test. The medium values and the standard deviation (SD) of the BMI were calculated, along with the percentage of fat mass, globally, by gender and age. A gender comparison was carried out with the *t*-test for independent samples. Furthermore, the size of the effect was calculated using Cohen’s *d* (Cohen, 1988).

In addition, the prevalence of overweight and obesity were calculated, by gender and age for the three references. The significant differences between references were calculated (Franklin, 2007) as well as the degree of concordance between each pair using the kappa coefficient (Cohen, 1960; Landis and Koch, 1977; Cerda and Villarroel, 2008). Traditionally, values <0 indicate no agreement, 0–0.20 slight, 0.21–0.40 fair, 0.41–0.60 moderate, 0.61–0.80 substantial, and 0.81–1 almost perfect agreement. Finally, the comparison of the prevalence of overweight and obesity between gender was carried out using the chi-square test (χ^2^). The significant value used was *p* < 0.05. The statistical package SPSS-22.0 (Statistical Package for the Social Sciences) and Microsoft Office Excel were used to carry out the analysis.

## Results

Table 1 shows the medium values and SD corresponding to the BMI and the percentage of fat mass of the 1000 children and adolescents from the sample. The results are shown organized in function to age and gender (Supplementary Figures S1, S2).

**Table 1:** Comparison of means of BMI and FM% (*N* = 1000).

Age (years)	BMI					FM %				
	Females	Males				Females	Males			
	(*n* = 512)	(*n* = 488)	Sig.	95% CI	d	(*n* = 512)	(*n* = 488)	Sig.	95% CI	d
7 (*n* = 57)	18.21 (3.08)	16.49 (2.19)	0.021^∗^	0.267 to 3.161	0.632	26.45 (5.79)	21.27 (4.05)	0.000^*^	2.477 to 7.884	1.116
8 (*n* = 24)	19.73 (2.92)	19.19 (3.24)	0.676	2.082 to 3.149	0.173	29.79 (5.93)	25.38 (6.42)	0.094	0.821 to 9.644	0.715
9 (*n* = 21)	19.79 (4.40)	19.66 (3.43)	0.947	3.703 to 3.947	0.030	26.08 (6.37)	24.86 (5.42)	0.659	4.465 to 6.897	0.202
10 (*n* = 79)	19.96 (5.46)	19.49 (2.90)	0.617	1.405 to 2.352	0.116	26.75 (5.75)	25.04 (6.13)	0.218	1.034 to 4.459	0.286
11 (*n* = 98)	19.83 (3.59)	20.24 (3.36)	0.575	1.842 to 1.027	0.116	25.72 (5.65)	23.99 (5.98)	0.150	0.638 to 4.098	0.299
12 (*n* = 77)	20.49 (2.98)	21.68 (3.10)	0.096	2.600 to 0.2183	0.394	25.52 (6.48)	22.95 (5.65)	0.079	0.307 to 5.439	0.416
13 (*n* = 76)	21.42 (5.01)	20.59 (3.88)	0.455	1.404 to 3.081	0.180	28.71 (8.54)	21.40 (7.50)	0.000^*^	3.539 to 11.065	0.986
14 (*n* = 88)	21.62 (3.32)	21.39 (3.20)	0.751	1.163 to 1.607	0.068	27.94 (7.29)	19.74 (6.95)	0.000^*^	5.172 to 11.222	0.871
15 (*n* = 53)	21.80 (3.53)	24.01 (4.86)	0.062	4.534 to 0.116	0.525	27.59 (6.27)	21.38 (7.44)	0.002^*^	2.428 to 9.991	1.162
16 (*n* = 82)	22.11 (3.63)	23.19 (5.35)	0.277	3.061 to 0.889	0.249	26.56 (6.94)	17.19 (8.28)	0.000^*^	5.982 to 12.755	0.933
17 (*n* = 77)	22.10 (2.66)	22.81 (2.76)	0.255	1.938 to 0.521	0.262	26.21 (6.18)	17.30 (6.01)	0.000^*^	6.144 to 11.681	0.937
18 (*n* = 153)	22.80 (4.74)	23.59 (3.71)	0.250	2.149 to 0.564	0.187	27.81 (7.04)	18.98 (6.60)	0.000^*^	6.653 to 11.011	0.646
19 (*n* = 115)	23.29 (5.20)	24.05 (4.54)	0.399	2.568 to 1.031	0.158	29.08 (7.77)	18.39 (6.51)	0.000^*^	8.054 to 13.329	0.755
Total (*n* = 1000)	21.34 (4.23)	21.76 (4.22)	0.122	0.938 to 0.111	0.098	27.15 (6.77)	20.71 (6.99)	0.000^*^	5.585 to 7.293	0.886


*Values are Mean (SD). ^∗^Significant differences between sexes.*

It is noteworthy that girls show higher medium values of fat mass than boys (*p* < 0.0001; *d* = 0.886). The fat mass in the female gender was higher in all age groups, showing significant differences at 7 years of age and from 13 to 19 years of age.

Tables 2, 3 show the prevalence of “overweight/excess fat” and “obesity” by gender, age and globally by the three references which have been studied: WHO, IOTF, CGF. They also show the significant differences found between references. Moreover, Supplementary Figures S3–S8 show graphically the different prevalences found when classifying the sample with the 3 references used.

**Table 2:** Prevalence (%) of overweight and obesity of females by age (*N* = 512).

Age	N	Overweight			Obesity			Overweight + Obesity		
		BMI WHO	BMI IOTF	FM % CGF	BMI WHO	BMI IOTF	FM % CGF	BMI WHO	BMI IOTF	FM% CGF
Females	512	22.3	21.9	17.6	11.3^3^	8.0^3^	18.2^1.2^	33.6	29.9^3^	35.8^2^
7	31	32.3	22.6	25.8	22.6	22.6	35.5	54.9	45.2	61.3
8	12	16.7	25.0	8.3	50	41.7	58.3	66.7	66.7	66.6
9	13	30.8	38.5	23.1	23.1	15.4	23.1	53.9	53.9	46.2
10	31	29.0	29.0	12.9	19.4	12.9	22.6	48.4	41.9	35.5
11	59	33.9^3^	30.5	16.9^1^	13.6	6.8	10.2	47.5^3^	37.3	27.1^1^
12	47	25.5	29.8	14.9	10.6	4.3	12.8	36.1	34.1	27.7
13	27	18.5	25.9	7.4	22.2	11.1^3^	33.3^2^	40.7	37.0	40.7
14	46	23.9	21.7	17.4	6.5	4.3^3^	17.4^2^	30.4	26.0	34.8
15	28	21.4	21.4	35.7	7.1	3.6	10.7	28.5	25.0	46.4
16	51	15.7	15.7	5.9	5.9	5.9	15.7	21.6	21.6	21.6
17	39	17.9	15.4	25.6	0.0	0.0	5.1	17.9	15.4	30.7
18	75	16.0	13.3	18.7	4.0^3^	4.0^3^	14.7^1.2^	20.0	17.3^3^	33.4^2^
19	53	15.1	17.0	18.9	11.3	9.4	22.6	26.4	26.4	41.5


*BMI, body mass index; FM %, fat mass percentage; WHO, world health organization; IOTF, international obesity task force; CGF, child growth foundation. Significant differences between references (*p* < 0.05) indicated with superindex: WHO = 1; IOTF = 2; CGF = 3.*

**Table 3:** Prevalence (%) of overweight and obesity of males by age (*N* = 488).

Age	N	Overweight			Obesity			Overweight + Obesity		
		BMI WHO	BMI IOTF	FM % CGF	BMI WHO	BMI IOTF	FM % CGF	BMI WHO	BMI IOTF	FM % CGF
Males	488	25.8	26.4	22.1	15.4^2.3^	8.4^1.3^	21.9^1.2^	41.2^2^	34.8^1.3^	44^2^
7	26	11.5	15.4	30.8	15.4	7.7	23.1	26.9^3^	23.1^3^	53.9^1.2^
8	12	25.0	33.3	25.0	33.3	25.0	41.7	58.3	58.3	66.7
9	8	37.5	37.5	25.0	25.0	12.5	37.5	62.5	50.0	62.5
10	48	35.4	39.6	33.3	27.1^2^	4.2^1.3^	25.0^2^	62.5	43.8	58.3
11	39	33.3	33.3	33.3	23.1^2^	5.1^1^	17.9	56.4	38.4	51.2
12	30	50.0	43.3	36.7	23.3	10.0	20.0	73.3	53.3	56.7
13	49	24.5	20.4	24.5	12.2	8.2	20.4	36.7	28.6	44.9
14	42	33.3	35.7^3^	16.7^2^	11.9^2^	0.0^1.3^	21.4^2^	45.2	35.7	38.1
15	25	32.0	32.0	24.0	20.0	20.0	32.0	52.0	52.0	56.0
16	31	12.9	12.9	16.1	12.9	12.9	16.1	25.8	25.8	32.2
17	38	21.1	21.1	10.5	5.3	5.3	10.5	26.4	26.4	21.0
18	78	20.5	20.5	14.1	9.0^3^	7.7^3^	24.4^1.2^	29.5	28.2	38.5
19	62	16.1	19.4	16.1	11.3	11.3	21.0	27.4	30.7	37.1


*BMI, body mass index; FM %, Fat mass percentage; WHO, world health organization; IOTF, international obesity task force; CGF, child growth foundation. Significant differences between references (*p* < 0.05) indicated with superindex: WHO = 1; IOTF = 2; CGF = 3.*

Significant differences (*p* < 0.05) were found in the prevalence of overweight and obesity between the three classifications. The total prevalence of overweight and obesity was of 32.3% according to IOTF, 37.3% according to WHO and 39.8% according to CGF. The WHO-IOTF concordance was substantial (kappa = 0.793), meanwhile the WHO-CGF (kappa = 0.504) and IOTF-CGF (kappa = 0.447) concordances were moderate.

The χ^2^ test showed that there is no statistical significance when making a global comparison of the prevalence of overweight and obesity in function to gender. However, it was observed that male demonstrate a greater prevalence independent of the classification used.

## Discussion

The three classifications used found a high prevalence of overweight and obesity in the studied sample. There were significant differences between the classifications: 32.3% according to IOTF, 37.3% according to WHO and 39.8% according to CGF. The WHO-IOTF concordance was substantial (kappa = 0.793), meanwhile the WHO-CGF (kappa = 0.504) and IOTF-CGF (kappa = 0.447) concordances were moderate.

The results of this study contrast with those found by Espín Ríos et al. (2013) who evaluated the BMI of 178,894 students (91,517 boys and 87,377 girls) of ages 2 to 14 in the region of Murcia (Spain), classifying them according to the criteria provided by the WHO and IOTF. Espín Ríos et al. (2013) found a greater prevalence of overweight and obesity in girls in contrast to the present study: 42.1% according to the WHO and 33.2% according to IOTF, in comparison to the 33.6 and 29.9% in this study. In boys, they observed a prevalence of 45.2% according to the WHO and 30.9% according to IOTF, in comparison to the 41.2 and 34.8% in this study. In terms of both genders, there was a prevalence of 43.7% according to the WHO and of 32% according to IOTF, percentages that somewhat differ to the current study: 37.3 and 32.3%, respectively. These observed discrepancies may be explained by differences in samples sizes and the different populations studied (Spain and Spain/Italy). For example, Spain and Italy are countries of Southern Europe with a similar Mediterranean diet, however, the percentage of total fat in the diet of Spanish adolescents has been found to be higher than in the diet of Italian adolescents (Cruz, 2000).

The National Health Survey 2011–2012 carried out by the National Institute of Statistics “INE” (Spain), in children and adolescents aged 2 to 17 years (3,580,100 female and 3,883,500 male), determined the BMI of all the participants and obtained the following percentages for overweight, obesity and excess weight: girls (16.94% overweight, 9.56% obesity, 26.50% excess weight), boys (19.46% overweight, 9.57% obesity, and 29.03% excess weight). The percentage of excess weight obtained by the Instituto Nacional de Estadística [INE] (2012) in girls (26.50%) is lower than those obtained in the present study (33.6% according to the WHO, 29.9% according to IOTF, and 35.8% according to CGF). Moreover, in boys the percentage of excess weight which was obtained by the INE (29.03%) is lower than the percentages obtained in the present study (41.2% according to the WHO, 34.8% according to IOTF, and 44% according to CGF). When only children and adolescents from the region of Murcia were considered, the data of the INE determined the following percentages for overweight, obesity and excess weight: girls (9.77% overweight, 16.75% obesity, 26.52% excess weight), boys (19.16% overweight, 9.46% obesity and 25.62% excess weight). The percentage of excess weight obtained in Murcia by the Instituto Nacional de Estadística [INE] (2012) in girls is lower than those obtained in the present study (33.6% according to the WHO, 29.9% according to IOTF, and 35.8% according to CGF). Moreover, in boys the percentage of excess weight which was obtained in Murcia by the INE (25.62%) is lower than the percentages obtained in this study (41.2% according to the WHO, 34.8% according to IOTF, and 44% according to CGF). This suggests that excess weight is increasing in the region of Murcia, Spain.

In the study by the National Institute of Statistics “ISTAT” (Italy) in 2010, in those aged 6 to 17 years (3,368,000 female and 3,558,000 male), the BMI of the participants was determined, and the following percentages of excess weight were obtained, according to IOTF criteria: 23.2% of excess weight in girls and 28.9% of excess weight in boys. The percentage of excess weight obtained by Instituto Nacional de Estadística [INE] (2012) for girls (23.2%) was lower than the estimate obtained in this study by IOTF (29.9%). Moreover, in boys the percentage of excess weight obtained by the ISTAT (28.9%) is lower than the percentage obtained in the present study by the IOTF (34.8%). When only children and adolescents from the regions of Lazio and Calabria were considered, the data of the ISTAT determined the following percentage for excess weight: 27.0% of excess weight in Lazio and 30.4% of excess weight in Calabria. This suggests that excess weight is increasing in the regions of Lazio and Calabria.

Previous studies (Wang and Wang, 2002; Shields and Tremblay, 2010; Espín Ríos et al., 2013; Bergel et al., 2014; López-Sánchez et al., 2015) with different samples (schoolchildren from Ibero-American countries, Canada, United States, Russia and China) also found important differences when using different classifications to evaluate the prevalence of overweight and obesity according to BMI, in the same way as the current study. However, the present study also found significant differences with the cut-off points for fat mass, which is why it is essential to specify always the methods and references used until an agreement is reached.

The main strength of the present study is the comparison between cut-off points for BMI and fat mass in a sample of children and adolescents residing in Southern Europe. However, the present study is not without limitations, the sample was composed of 1000 children and adolescents from distinct geographic areas/countries and, although all the recommendations for an adequate analysis of electric bioimpedance were followed, the electrical bioimpedance is not the gold standard method to evaluate fat mass.

## Conclusion

The present findings suggest that there is a high prevalence of overweight and obesity in children and adolescents residing in Southern Europe. However, prevalence estimates of overweight and obesity differ by methods and reference cut points. Higher prevalence was obtained with the classification of CGF (fat mass), followed by the classifications of WHO and IOTF (BMI).

## Practical Applications

A precise definition of overweight and obesity is needed, as well recommended methods for evaluation, and accurate cut-off points. The authors of this study recommend the evaluation of overweight and obesity not only by BMI, but also through the percentage of fat mass, and to always specify the references used to classify the sample.

It would also be convenient to carry out regular assessments in schools, which could be carried out by the Physical Education teacher because of the direct connection between the subject and overweight and obesity. This simple practice would provide updated reference values in any geographical location.

Finally, due to the high prevalence of overweight and obesity found in this study, it would be interesting to carry out intervention programs in children and adolescents, through physical activity and a dietary improvement, to reduce their fat mass and BMI values and develop healthy lifestyle habits. To control the dietary habits during these intervention programs, researchers should evaluate regularly the quality of the diet of the children and adolescents participating in the intervention. Future researchers should also consider the psychological factors of motivation and perceived motor competence, and the family influence, when carrying out intervention programs to ensure that the children and adolescents will adhere to any program put in place.

## Author Contributions

All authors listed have made a substantial, direct and intellectual contribution to the work, and approved it for publication.

## Conflict of Interest Statement

The authors declare that the research was conducted in the absence of any commercial or financial relationships that could be construed as a potential conflict of interest.
