# Supplementary material for: Body Composition in Children and Adolescents Residing in Southern Europe: Prevalence of Overweight and Obesity According to Different International References
Source: Front Physiol. 2019 Feb 19;10:130. doi: 10.3389/fphys.2019.00130 (PMC6390201; doi:10.3389/fphys.2019.00130)
Supplement: Supplementary file 1 [file Table_1.DOCX]

Supplementary Material

**Body Composition in children and adolescents residing in Southern Europe:**

**Prevalence of overweight and obesity according to different international references**

**Guillermo Felipe López-Sánchez^1^*, Maurizio Sgroi^2^, Stefano D'Ottavio^2^, Arturo Díaz-Suárez^1^, Sixto González-Víllora^3^, Nicola Veronese^4^, Lee Smith^5^**

*** Correspondence:** Guillermo Felipe López-Sánchez: gfls@um.es

| *Figure S1. BMI according to gender and age* | *Figure S2. FM % according to gender and age* |
| --- | --- |
