# Supplementary material for: Body Composition in Children and Adolescents Residing in Southern Europe: Prevalence of Overweight and Obesity According to Different International References
Source: Front Physiol. 2019 Feb 19;10:130. doi: 10.3389/fphys.2019.00130 (PMC6390201; doi:10.3389/fphys.2019.00130)
Supplement: Supplementary file 2 [file Table_2.DOCX]

Supplementary Material

**Body Composition in children and adolescents residing in Southern Europe:**

**Prevalence of overweight and obesity according to different international references**

**Guillermo Felipe López-Sánchez^1^*, Maurizio Sgroi^2^, Stefano D'Ottavio^2^, Arturo Díaz-Suárez^1^, Sixto González-Víllora^3^, Nicola Veronese^4^, Lee Smith^5^**

*** Correspondence:** Guillermo Felipe López-Sánchez: gfls@um.es

*Figure S3.* Overweight in females *Figure S4.* Overweight in males

*Figure S5.* Obesity in females *Figure S6.* Obesity in males

*Figure S7.* Overweight + Obesity in females *Figure S8.* Overweight + Obesity in males
